# C-reactive protein can predict dose intensity, time to treatment failure and overall survival in HCC treated with lenvatinib

**DOI:** 10.1371/journal.pone.0244370

**Published:** 2020-12-22

**Authors:** Tsuguru Hayashi, Michihiko Shibata, Shinji Oe, Koichiro Miyagawa, Yuichi Honma, Masaru Harada

**Affiliations:** Third Department of Internal Medicine, University of Occupational and Environmental health, Fukuoka, Japan; National Yang-Ming University, TAIWAN

## Abstract

**Background and aim:**

Lenvatinib has become a first line treatment for unresectable hepatocellular carcinoma (HCC). However, continued administration is impossible in many patients due to treatment resistance and severe adverse events. This study aimed to identify predicting factors to select patients likely to benefit from lenvatinib treatment.

**Methods:**

We retrospectively analyzed 53 patients who were treated with lenvatinib for unresectable HCC. They were divided to two groups; low C-reactive protein (CRP) group with pretreatment serum CRP level < 1.0 mg/dL and high CRP group with serum CRP level ≥ 1.0 mg/dl. Overall survival (OS), total amount administered, and period of treatment were compared between the two groups.

**Results:**

The high CRP group showed a significantly poorer OS than the low CRP group (0.0% vs 71.5%/ 1year, p < 0.01). Multivariate analyses revealed that high CRP was a significant negative factor for OS (HR: 7.69, 95% confidence interval: 2.43–24.3, p < 0.001), and this result was independent of Child-Pugh score and existing tumor factors. Relative dose intensity at 8 weeks was lower (p = 0.01) and time to treatment failure was shorter (P < 0.001) in the high CRP group.

**Conclusions:**

CRP level was associated with OS in HCC patients treated with lenvatinib. CRP could be a useful marker to identify patients most likely to benefit from lenvatinib treatment.

## Introduction

Hepatocellular carcinoma (HCC) is the fifth most common tumor worldwide and the second most common cause of cancer-related death [1]. Although the prognosis of patients with HCC has improved considerably with the development of treatments such as surgical resection, local ablation and transcatheter arterial chemoembolization (TACE), the prognosis of those with advanced HCC remains poor. Furthermore, despite HCC screening systems [2], many cases are diagnosed at an advanced stage [3].

In addition to sorafenib, several drugs such as lenvatinib, regorafenib, ramucirumab and cabozantinib, have shown some effect on unresectable HCC in recent years [4–9]. In particular, lenvatinib demonstrated a high antitumor effect in the REFLECT trial [4]. The objective control rate and disease control rate of lenvatinib were 40.6% and 73.8%, respectively, and those levels were higher than those with sorafenib. As a result, lenvatinib is now used as a first-line systematic treatment for advanced unresectable HCC in Japanese clinical practice [10,11]. However, many patients are unable to continue lenvatinib for a long period due to tumor progression and adverse events (AEs), such as fatigue, diarrhea and decreased appetite. Therefore, there is an urgent need to identify the patients most likely to benefits from lenvatinib in terms of antitumor effects and AEs before treatment.

Recently, several studies have shown that C-reactive protein (CRP) was associated with the prognoses of various malignancies [12–16]. In HCC, CRP was associated with prognoses after liver resection, TACE, liver transplantation and sorafenib [17–22]. The mechanism behind this association between CRP and cancer prognosis remains largely unknown. However, previous studies have shown that aggressive cancer behavior produced a detrimental inflammatory response, which lead to elevated serum CRP level. Furthermore, elevated inflammatory response may promote the progression of cancer cells [23]. Interleukin-6 (IL-6) is a proinflammatory cytokine. IL-6 activates signal transducer and activator of transcription 3 (STAT3), which is crucial for proliferation of cancer cells. In addition, STAT3 has various effects on cancer cell activity, such as angiogenesis, metastasis, and inhibition of cancer cell apoptosis [24,25]. Further, IL-6 affects hepatocytes directly and produces CRP [26]. Therefore, serum IL-6 level reflects CRP. In fact, elevated IL-6 and CRP are associated with a high risk of HCC. However, the relationship between CRP levels and effect of lenvatinib is unknown.

In this study, we investigated the relationship between the prognosis and the amount and period of lenvatinib treatment.

## Material and methods

### Patients

This retrospective study enrolled 53 consecutive patients with unresectable HCC who had been treated with lenvatinib at our university hospital from May 2018 to April 2020. The diagnosis of HCC was based on histology or radiological findings, such as contrast enhanced computed tomography (CT) or contrast enhanced magnetic resonance imaging (MRI). Barcelona Clinic Liver Cancer (BCLC) stage was used for evaluation of HCC staging. This study was approved by the institutional ethical board in accordance with the Declaration of Helsinki (Ethics Committee of Medical Research, University of Occupational and Environmental Health, Japan; H29-078). All data were anonymized before analyses and ethics committee waived the requirement for informed consent because of the retrospective observational study. The data range during which patients medical record was accessed was from May 2018 to July 2020. The source of the medical records analyzed was hospital.

### Lenvatinib treatment

Lenvatinib (Lenvima^®^; Eisai Co., Ltd., Tokyo, Japan) was orally administered to patients with unresectable HCC. The initial dose of lenvatinib was based on body weight as set in the guidelines for administration of lenvatinib, 12 mg once daily for those over 60 kg and 8 mg once daily for those under 60 kg body weight. Measurement of blood samples and multiphase-multidirector CT imaging were performed before and every month after starting lenvatinib in all patients. Response to lenvatinib was evaluated by image findings according to the modified Response Evaluation Criteria in Solid Tumors (mRECIST). Lenvatinib was reduced or interrupted when unacceptable AEs occurred or in case of clinical tumor progression.

### Survival analysis

We classified the patients with a serum CRP of level 1.0 mg/dL or more into the high CRP group, and those with CRP less than 1.0 mg/dL into the low CRP group. The cut-off value of CRP was based on the results of previous studies [17–22,27]. We compared age, sex, body mass index (BMI), etiology, liver function and tumor factors between the two groups at the start of lenvatinib treatment.

The endpoint of this study was overall survival (OS), which was time from the initial administration of lenvatinib to death from any cause or last follow-up. We also evaluated time to tumor progression (TTP), time to treatment failure (TTF), time to decompensation (Child-Pugh grade B or C), time to liver related events and relative dose intensity (RDI). TTF was time from the initial treatment to discontinuation of lenvatinib. Liver related events were defined as hepatic encephalopathy, ascites retention and acute on chronic liver failure. RDI was calculated at 4 and 8 weeks.

### Adverse events

AEs related to lenvatinib treatment were assessed using the National Cancer Institute Common Terminology Criteria for Adverse Events, version 4.0. The highest grade for each AE during the observation period was recorded. Severe AE was defined as AE 3 and 4.

### Statistical analysis

All categorical variables were analyzed using χ^2^-test or Fisher’s exact test, and continuous variables were compared using Mann-Whitney’s U test. P value < 0.05 was considered statistically significant. OS, TTP, TTF, time to decompensation and liver related events were evaluated based on the Kaplan-Meier curve, and differences between the two groups were assessed using the log-rank test. A Cox proportional hazards model was used to determine the factors associated with OS. The cut off values that were used in univariate and multivariate analyses were medians of all patients. All variables with p value < 0.05 from the univariate analyses were included in the multivariate analyses using backward elimination method. Change of CRP after lenvatinib treatment was compared using the Wilcoxon single-rank sum test. Continuous numeric variables were expressed as median and interquartile range (IQR). All statistical analyses were performed using the Statistical Package for the Social Science (SPSS) version 25 (SPSS Inc., Chicago, IL, USA) and Easy R (EZR) version 1.29 (Saitama Medical center, Jichi Medical University, Saitama, Japan), and graphical use interface for R (The R Foundation for Statistical Computing, Vienna, Austria).

## Results

### Patients characteristics

Patient characteristics were listed in Table 1. The median age was 73 years and 79.2% were male. Median BMI was 23.7 kg/m^2^. Initial dose of lenvatinib was 12 mg in 22 patients and 8 mg in 31 patients. Four patients were started at a reduced dose because of advanced age (>80 years) and poor performance status (≧2). All patients at baseline were Child-Pugh grade A. Patients with Child-Pugh scores of 5 and 6 points numbered 30 and 23, respectively. BCLC Stage A, B, and C were 5, 23, and 25, respectively. Eighteen patients had extrahepatic metastases, and 10 had major vascular invasion. The high CRP group contained 17 patients, and the low CRP group contained 36. The high CRP group had lower serum albumin, a higher rate of major vascular invasion and higher alfa-fetoprotein (AFP) and des-gamma-carboxy prothrombin (DCP) level compared to the low CRP group. No patients with BCLC stage A and B received locoregional therapy such as radiofrequency ablation (RFA) and TACE during lenvatinib treatment. We confirmed that all patients did not have infection and collagen disease, such as rheumatoid arthritis.

**Table 1:** Baseline characteristics.

	ALL	High CRP group	Low CRP group	P-value
N	53	17	36	
Age (IQR), years	73 (67–77)	74 (68–78)	72(67–76)	0.26
Male (%)	42 (79.2)	14 (82.4)	28 (77.8)	1.00
Body Weight (IQR), kg	61.0 (54.0–67.6)	61.0 (54.6–67.6)	60.9 (53.8–66.5)	0.89
BMI (IQR), kg/m^2^	23.7 (22.0–25.7)	24.4 (22.5–25.6)	23.3 (21.9–26.2)	0.98
Initial dose of lenvatinib 8/12 mg	31/22	11/6	20/16	0.57
Etiology, HBV/HCV/ NBNC	7/15/21	3/6/8	4/12/20	0.72
Child-Pugh score, 5/6 points	30/23	3/14	27/9	<0.001
Albumin (IQR), g/dL	3.7 (3.3–3.9)	3.2 (3.1–3.4)	3.8 (3.6–4.0)	<0.001
Bilirubin (IQR), mg/dL	0.7 (0.6–1.0)	0.7 (0.5–0.9)	0.7(0.6–1.0)	0.71
Prothrombin time (IQR), %	88.1 (80.6–95.6)	88.1 (81.4–95.5)	89.6 (80.2–95.9)	0.94
CRP (IQR), mg/dL	0.44 (0.16–1.48)	1.89 (1.5–3.22)	0.21 (0.10–0.45)	<0.001
BCLC Stage, A/B/C	5/23/25	0/3/14	5/20/11	0.002
Major vascular invasion (%)	10 (18.9)	7 (41.2)	3 (8.3)	0.01
Extra hepatic metastasis (%)	18 (34.0)	10 (58.8)	8 (22.2)	0.02
AFP (IQR), ng/mL	189 (10–2212)	495 (34–9671)	58.5 (8–444)	0.04
DCP (IQR), mAU/mL	235 (43–1369)	1369 (635–5889)	113 (35–633)	0.001

Abbreviation: AFP, alfa fetoprotein; BCLC, barcelona clinic liver cancer; BMI, body mass index; CRP, c-reactive protein; DCP, des-gamma-carboxy prothrombin, NBNC, non-HBV and non-HCV. Continuous numeric variables were expressed as median and interquartile range (IQR).

### Factors associated with OS

**Fig 1:**
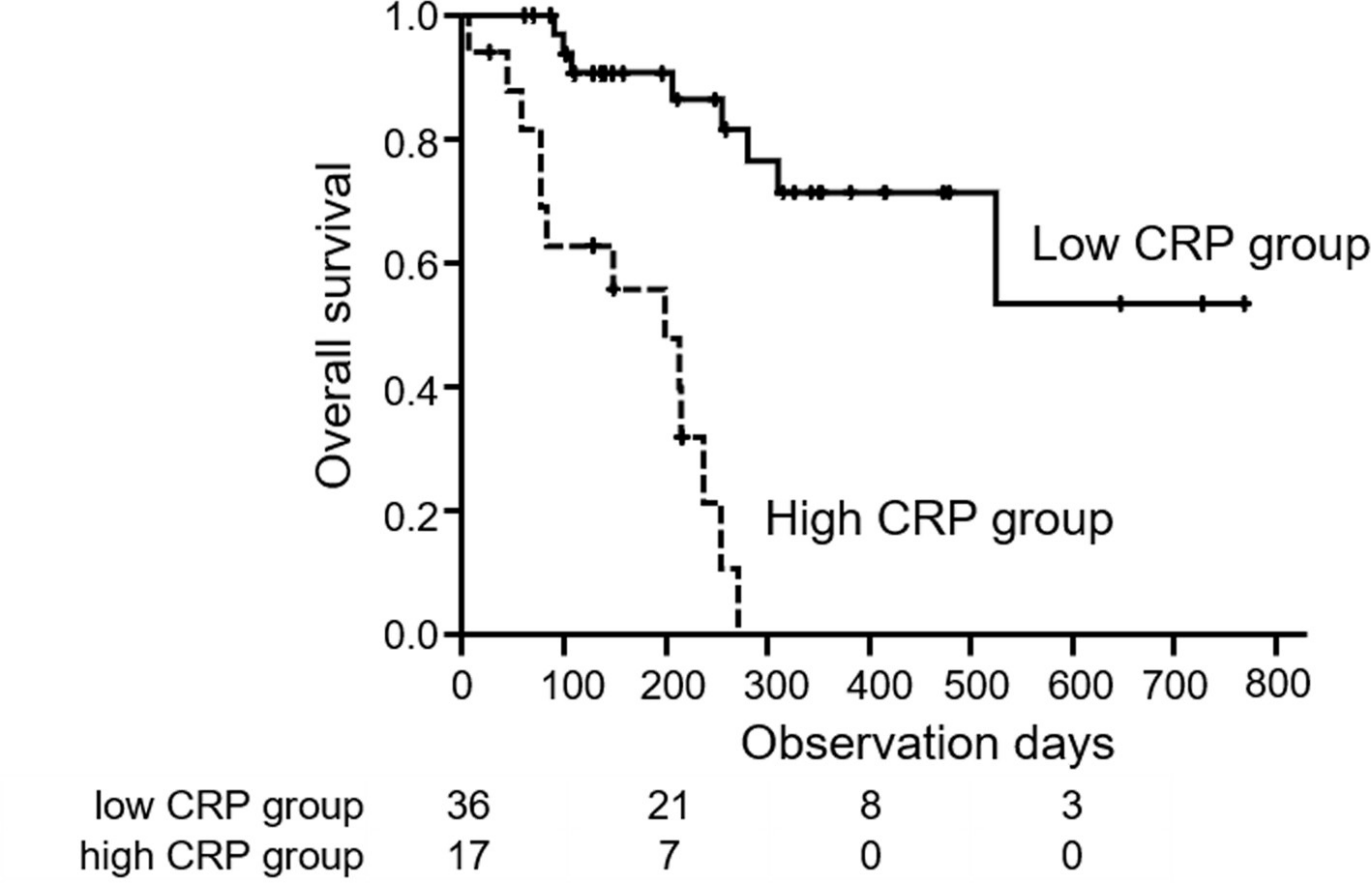
Overall survival with unresectable HCC treated with lenvatinib in high and low CRP groups. The low CRP group (solid line) showed a significantly better prognosis than the high CRP group (dotted line) (71.5% vs 0.0%/ 1year, log-rank test; p < 0.01).

The median observation period after starting lenvatinib was 207 days. The median survival time (MST) in all patients was 311 days. OS of the high CRP group was significantly shorter than that of the low CRP group (0.0% vs 71.5%/ 1year, p < 0.01) (Fig 1). Prognostic factors associated with OS were assessed by univariate and multivariate analyses. According to univariate analysis, Child-Pugh score 6 points (Hazard ratio (HR): 5.10, 95% confidence interval (CI): 1.95–13.3, p < 0.001), albumin < 3.7 g/dL (HR: 9.01, 95% CI: 2.58–31.4, p < 0.001), BCLC stage C (HR: 5.44, 95% CI: 1.82–26.2, p = 0.002), high CRP (HR: 10.9, 95% CI: 3.68–32.0, p < 0.001), major vascular invasion (HR: 6.67, 95% CI: 2.74–16.3, p < 0.001) and DCP > 235 mAU/mL (HR: 4.90, 95% CI: 1.77–13.6, p = 0.002) were significantly associated with OS. In multivariate analysis, high CRP (HR: 7.69, 95% CI: 2.43–24.3, p < 0.001) and major vascular invasion (HR: 3.87, 95% CI: 1.50–10.0, p = 0.005) were significant independent factors associated with OS (Table 2).

**Table 2:** Univariate and multivariate analysis to determine the factors associated with overall survival.

	Univariate			Multivariate		
	HR	95%CI	P-value	HR	95%CI	P-value
Age (>73)	0.82	0.34–1.96	0.66			
Male	0.60	0.22–1.66	0.33			
Body Weight (>61)	0.86	0.36–2.04	0.73			
BMI (>23.7)	1.70	0.70–4.09	0.24			
Initial dose of lenvatinib (12)	0.78	0.32–1.91	0.59			
Etiology (HCV)	1.51	0.48–4.76	0.49			
Etiology (NBNC)	0.40	0.11–1.42	0.16			
Child-Pugh score 6	5.10	1.95–13.3	<0.001	2.63	0.98–7.0	0.054
Albumin (<3.7)	9.01	2.58–31.4	<0.001	1.93	0.32–11.8	0.48
Bilirubin (>0.7)	1.46	0.58–3.63	0.41			
Prothrombin time (<88)	1.07	0.45–2.52	0.88			
BCLC stage (C)	5.44	1.82–16.2	0.002	1.44	0.34–6.10	0.62
CRP (>1.0)	10.9	3.68–32.0	<0.001	7.69	2.43–24.3	<0.001
Major vascular invasion	6.67	2.74–16.3	<0.001	3.87	1.50–10.0	0.005
Extra hepatic metastasis	2.02	0.86–4.78	0.11			
AFP (>189)	2.36	0.94–5.92	0.07			
DCP(>235)	4.90	1.77–13.6	0.002	2.76	0.80–9.52	0.11

Abbreviation: AFP, alfa fetoprotein; BCLC, barcelona clinic liver cancer; BMI, body mass index; CRP, c-reactive protein; DCP, des-gamma-carboxy prothrombin.

### Relative dose intensity and treatment period of lenvatinib

**Fig 2:**
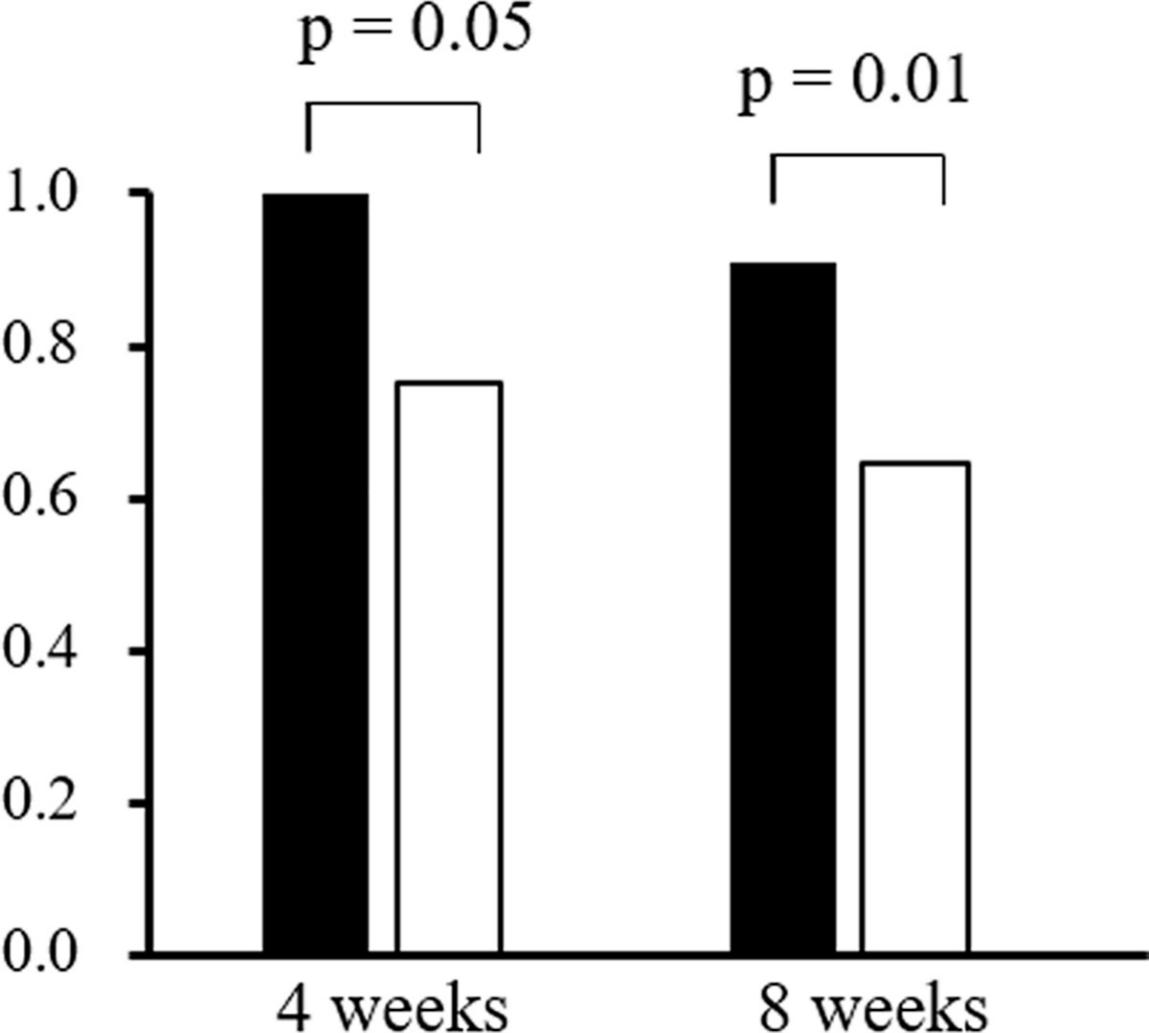
Relative dose intensity (RDI) of lenvatinib at 4 and 8 weeks. White bar showed the median value in the high CRP group and the black bar shows that of the low CRP group. RDI in the high and low CRP groups was 0.75 and 1.00 at 4 weeks, and 0.65 and 0.91 at 8weeks, respectively. Although the p value of RDI at 4 weeks was 0.054, it was 0.01 at 8 weeks by Mann-Whitney’s U test.

**Fig 3:**
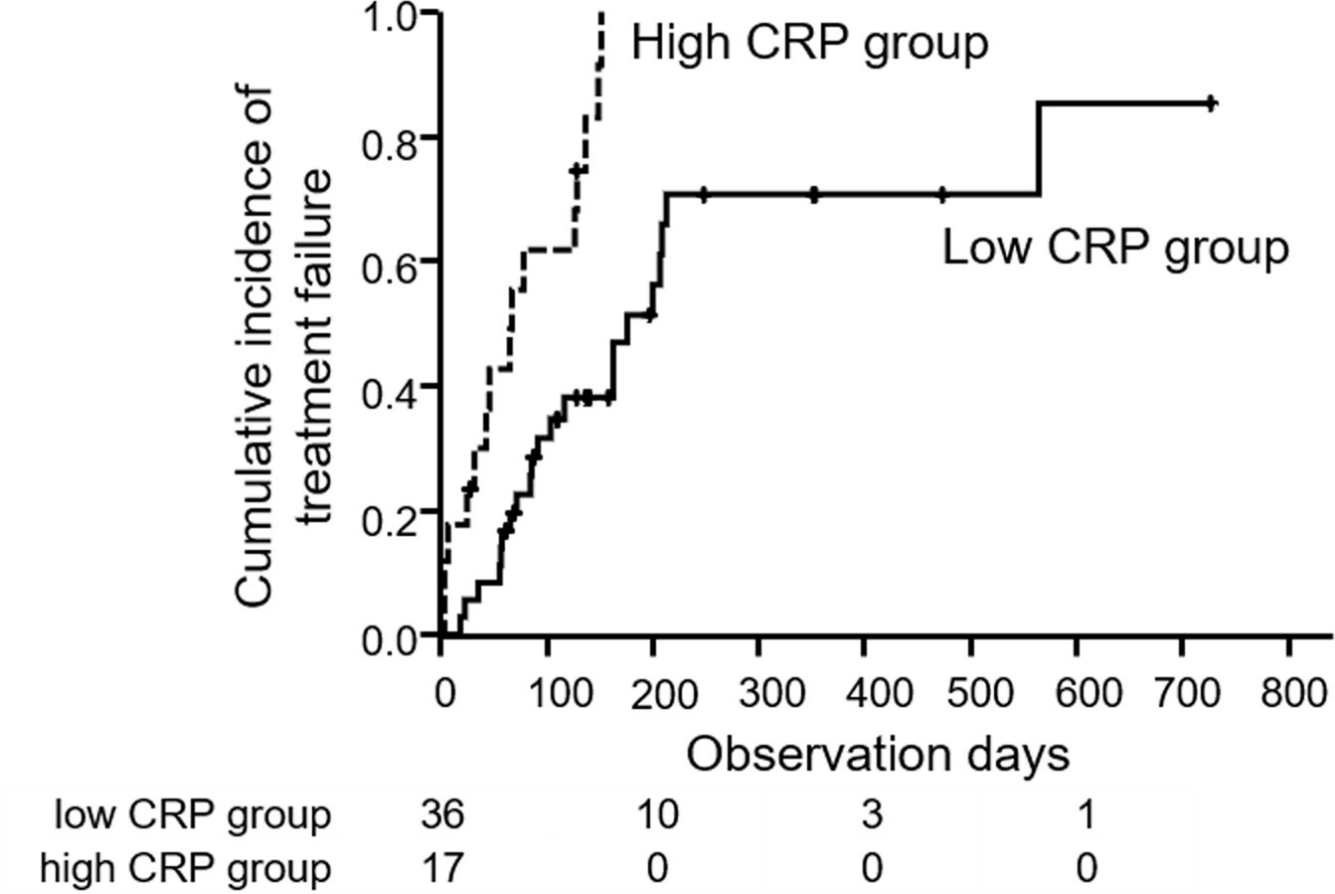
Time to treatment failure (TTF) in high and low CRP groups. Median TTFs in high CRP group (dotted line) and low CRP group (solid line) were 67 and 176 days, respectively (log-rank test; p < 0.001).

The median RDIs in all patients were 1.0 and 0.83 at 4 and 8 weeks, respectively. RDIs in the high and low CRP groups were shown in Fig 2. Median RDI at 4 weeks was 1.00 in the low CRP group and 0.75 in the high CRP group (p = 0.054). At 8 weeks, the median RDI of the low CRP group (0.91) was significantly higher than that of the high CRP group (0.65, p = 0.01). Fig 3 showed the TTF. Median TTFs were 67 days in the high CRP group and 176 days in the low CRP group, and this difference was significant (p < 0.001). In univariate analysis, male, initial dose of lenvatinib (12mg), etiology (NBNC), Child-Pugh score 6, albumin < 3.7 g/dL, high CRP, major vascular invasion, AFP > 189 mAU/mL and DCP > 235 mAU/mL were independent factors that influence TTF significantly. In multivariate analysis, male, etiology (NBNC), albumin < 3.7 g/dL and high CRP were independent factors that influence TTF significantly (Table 3). Thirty five patients (66.0%) discontinued lenvatinib treatment. The reasons for lenvatinib discontinuation were AE (42.9%), tumor progression (42.9%) and liver decompensation (14.2%). There was no difference in high and low groups (AE: 50.0% vs 38.1%, tumor progression: 35.7% vs 47.6%, liver decompensation: 14.3% vs 14.3%, p = 0.89).

**Table 3:** Univariate and multivariate analysis to determine the factors associated with TTF.

	Univariate			Multivariate		
	HR	95%CI	P-value	HR	95%CI	P-value
Age (>73)	1.29	0.66–2.52	0.45			
Male	0.38	0.17–0.82	0.01	0.21	0.09–0.50	<0.001
Body Weight (>61)	0.87	0.45–1.67	0.67			
BMI (>23.7)	1.12	0.59–2.14	0.73			
Initial dose of lenvatinib (12)	0.49	0.24–0.99	0.047	1.24	0.51–3.03	0.64
Etiology (HCV)	0.82	0.31–2.15	0.07	0.68	0.23–2.00	0.48
Etiology (NBNC)	0.31	0.11–0.82	0.02	0.33	0.11–0.94	0.04
Child-Pugh score 6	3.20	1.65–6.21	<0.001	1.48	0.54–4.00	0.45
Albumin (<3.7)	3.62	1.79–7.29	<0.001	2.82	1.23–6.42	0.01
Bilirubin (>0.7)	1.14	0.59–2.23	0.69			
Prothrombin time (<88)	1.48	0.77–2.85	0.24			
BCLC stage (C)	1.26	0.66–2.40	0.49			
CRP (>1.0)	4.29	2.04–9.03	<0.001	3.36	1.41–8.00	0.006
Major vascular invasion	4.11	1.83–9.25	<0.001	1.04	0.38–2.81	0.95
Extra hepatic metastasis	0.96	0.49–1.86	0.89			
AFP (>189)	2.24	1.11–4.50	0.02	1.42	0.65–3.06	0.38
DCP(>235)	2.86	1.42–5.75	0.003	1.00	0.28–3.65	1.00

Abbreviation: AFP, alfa fetoprotein; BCLC, barcelona clinic liver cancer; BMI, body mass index; CRP, c-reactive protein; DCP, des-gamma-carboxy prothrombin.

### Response to lenvatinib and time to progression

**Fig 4:**
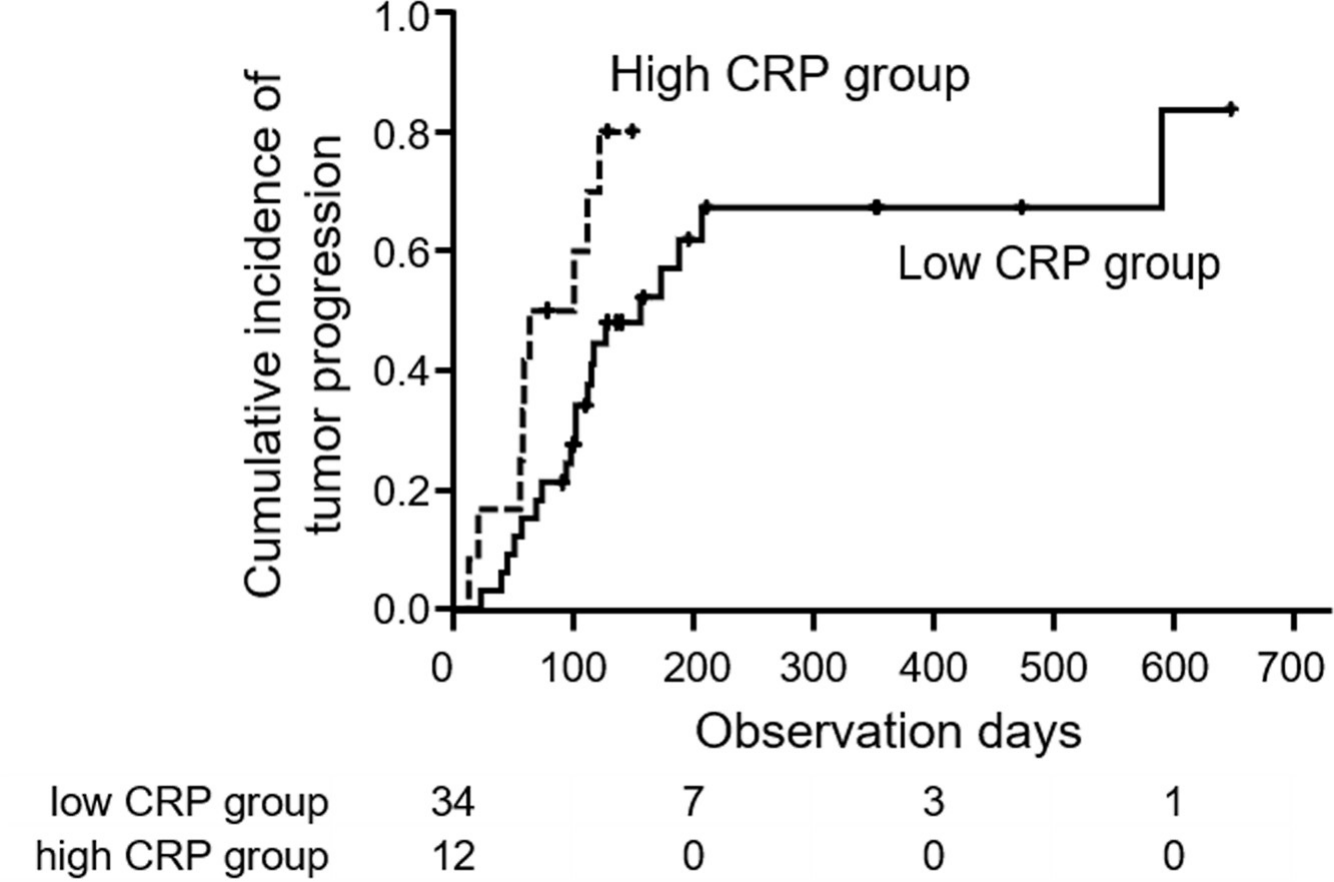
Time to progression (TTP) in high and low CRP groups. Median TTPs in high CRP group (dotted line) and low CRP group (solid line) were 83 and 156 days, respectively (log-rank test; p = 0.03).

Among the 53 patients, 46 received radiological evaluations and the rates of partial response (PR), stable disease (SD) and progressive disease (PD) were 39.1%, 26.1% and 34.8%, respectively. Seven patients were not evaluated because of early treatment failure. In high CRP group, PR, SD and PD was 8.3%, 25.0% and 66.7%, respectively. In low CRP, CRP group, PR, SD and PD was 50.0%, 26.5% and 23.5%, respectively. Objective control rate and disease control rate was significantly higher in low CRP group (p = 0.03 and 0.01, respectively). The median TTP in all patients was 117 days. The median TTP was significantly longer in the low CRP group than in the high CRP group (156 vs 83 days, p = 0.03) (Fig 4). In univariate analysis, Child-Pugh score 6, albumin < 3.7 g/dL and high CRP were independent factors that influence TTP significantly. In multivariate analysis, Child-Pugh score 6 was an independent factor that influence TTF significantly (Table 4).

**Table 4:** Univariate and multivariate analysis to determine the factors associated with TTP.

	Univariate			Multivariate		
	HR	95%CI	P-value	HR	95%CI	P-value
Age (>73)	0.98	0.47–2.03	0.95			
Male	0.54	0.19–1.48	0.23			
Body Weight (>61)	0.73	0.35–1.53	0.41			
BMI (>23.7)	0.98	0.46–2.07	0.96			
Initial dose of lenvatinib (12)	0.48	0.22–1.07	0.07			
Etiology (HCV)	0.68	0.21–2.21	0.52			
Etiology (NBNC)	0.42	0.14–1.30	0.13			
Child-Pugh score 6	3.36	1.53–7.41	0.003	3.36	1.53–7.41	0.003
Albumin (<3.7)	2.65	1.23–5.70	0.01	1.26	0.39–4.01	0.69
Bilirubin (>0.7)	1.06	0.50–2.27	0.87			
Prothrombin time (<88)	1.39	0.67–2.89	0.38			
BCLC stage (C)	0.76	0.36–1.62	0.48			
CRP (>1.0)	2.47	1.07–5.69	0.03	1.69	0.70–4.07	0.25
Major vascular invasion	2.52	0.98–6.50	0.06			
Extra hepatic metastasis	0.74	0.33–1.66	0.46			
AFP (>189)	1.52	0.70–3.26	0.29			
DCP(>235)	1.86	0.87–3.95	0.11			

Abbreviation: AFP, alfa fetoprotein; BCLC, barcelona clinic liver cancer; BMI, body mass index; CRP, c-reactive protein; DCP, des-gamma-carboxy prothrombin.

### Child-Pugh grade deterioration and liver related events

**Fig 5:**
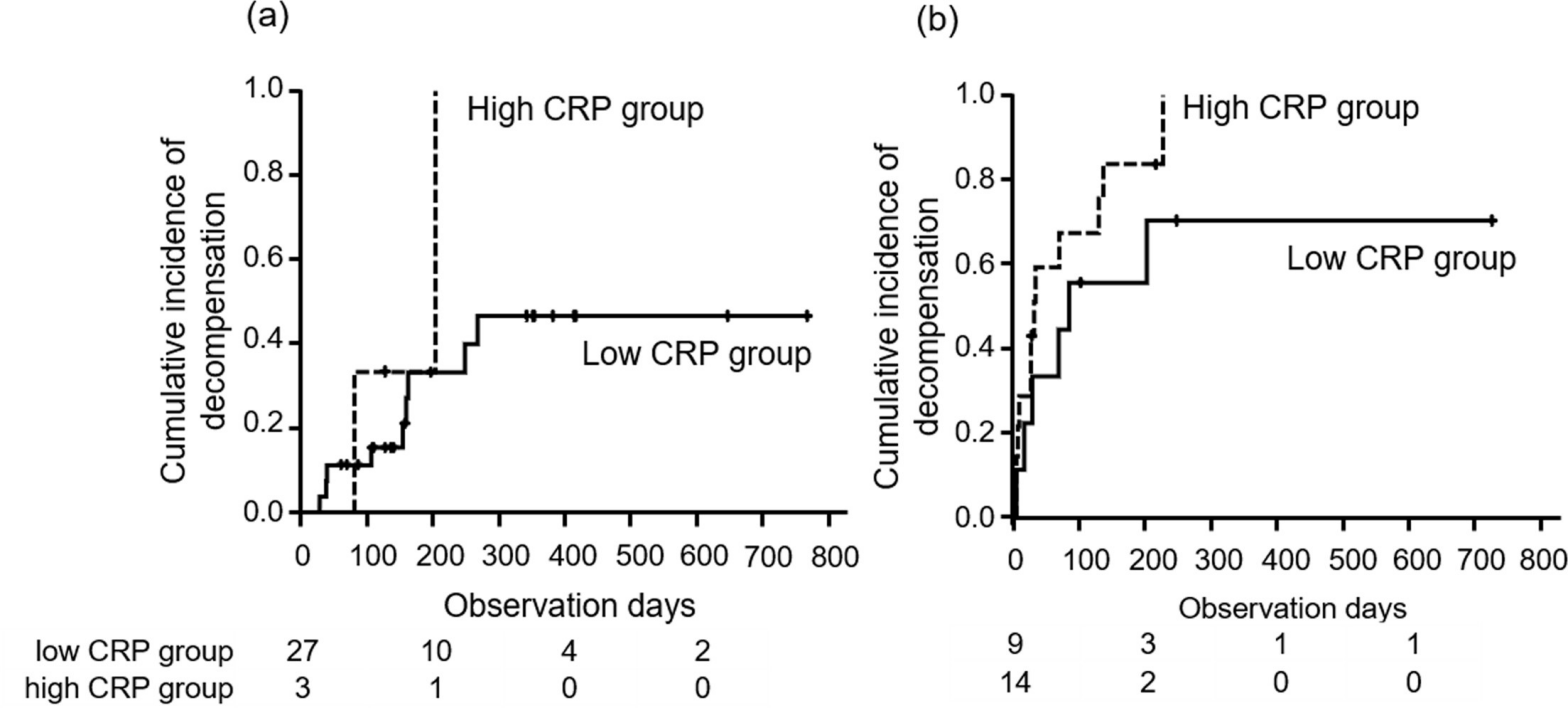
Time to decompensation stratified by baseline Child-Pugh score, 5 (a) and 6 (b). High and low CRP groups were expressed by dotted and solid lines, respectively. P values by log-rank tests for Child-Pugh score 5 and 6 were 0.17 and 0.22, respectively.

Cumulative incidences of decompensation in all patients were 11.3% and 26.7% at 4 and 8 weeks, respectively. The median time to decompensation did not differ between the two groups stratified by baseline Child-Pugh score 5 and 6 (Fig 5). In patients with a Child-Pugh score of 5 points, time to decompensation in the high and low CRP groups were not different (100% vs 46.5%/ 1year, p = 0.17). In those with a Child-Pugh score of 6, time to decompensation in the high and low CRP groups were not different (70.4% vs 100%/ 1year, p = 0.22).

Cumulative incidence of liver related events in two groups were stratified by Child-Pugh score 5 and 6. In patients with a Child-Pugh score of 5 points, time to liver related events in the high and low CRP groups were significantly different (34.9% vs 100%/ 1year, p = 0.003). In those with a Child-Pugh score of 6, time to liver related events in the high and low CRP groups were not different (73.3% vs 44.9%/ 1year, p = 0.72).

### Adverse events

Treatment-related AEs were shown in Table 5. There were no differences in the frequency of any AE between the two groups.

**Table 5:** Adverse events.

	High CRP group	Low CRP group	P-value
All adverse events n (%)	17 (100.0)	31 (86.1)	0.27
Fatigue n (%)	9 (52.9)	11 (30.6)	0.21
Decrease of appetite n (%)	10 (58.8)	14 (38.9)	0.29
Hypertension n (%)	5 (29.4)	12 (33.3)	1.00
Proteinuria n (%)	3 (17.6)	10 (27.8)	0.65
Palmar-plantar erythrodysesthesia n (%)	5 (29.4)	6 (16.7)	0.48
Diarrhea n (%)	4 (23.5)	9 (25.0)	1.00
Decrease of platelet counts n (%)	0 (0.0)	8 (22.2)	0.09
Hypothyroidism n (%)	3 (17.6)	15 (41.7)	0.16
Severe adverse events n (%)	4 (23.5)	4 (11.1)	0.44

### Change of CRP after administration

We also evaluated CRP change and radiological response to lenvatinib. Forty two patients were measured CRP at first imaging evaluations such as CT or MRI. Residual 11 patients were not measured or had infections. CRP elevated from 0.35 to 0.70 after lenvatinib treatment (n = 42, p = 0.01). In high CRP group, CRP did not change both in patients with PR and SD (from 1.79 to 1.86, p = 0.75) and in patients with PD (from 1.74 to 2.19, p = 0.55). However, in low CRP group, CRP increased in patients with PR and SD (from 0.22 to 0.26, p = 0.03), but not in patients with PD (from 0.19 to 0.96, p = 0.10) did not change.

## Discussion

This study indicated that CRP was the best predictor of OS in unresectable HCC patients treated with lenvatinib, and this finding was independent of Child-Pugh score and existing tumor factors. Although the incidences of AEs were similar regardless of CRP level, the low CRP group showed better RDI, TTF, and response to lenvatinib than the high CRP group. These results led to a statistically improved prognosis. Based on these findings, we consider this simple maker, CRP, to be the best indicator for lenvatinib in patients with unresectable HCC.

Systemic therapies are the main methods of treating advanced unresectable HCC. Such therapies have made rapid progress in recent years, however, the optimal use of the various drugs is still unknown. Therefore, the importance of individualized management of advanced HCC is increasing rapidly. Selection of the most appropriate drug for each patient will lead to improving the prognosis of advanced HCC. Several candidate biomarkers, such as plasma vascular endothelial growth factor (VEGF) and gene expression, have been reported to solve these problems [28–30]. However, these are costly and not readily available.

Lenvatinib is a tyrosine kinase inhibitor that targets VEGF receptors 1–3, fibroblast growth factor (FGF) receptors 1–4, platelet derived growth factor (PDGF) alpha, rearranged during transfection (RET) and Kit. The phase III REFLECT clinical trial showed that lenvatinib was not inferior to sorafenib in OS and had a better antitumor effect than sorafenib in patients with unresectable HCC [4]. However, lenvatinib related treatment-emergent AEs occurred in 75% of patients. Therefore, rates of drug interruption, dose reduction, and drug withdrawal were 40%, 37% and 9%, respectively. In real world clinical practice, AEs occurred frequently and only a limited number of patients could continue lenvatinib. However, new methods to cope with these AEs and continue lenvatinib have not been found. Therefore, it is important to select the patients who are suitable for lenvatinib treatment. It was also reported that RDI was correlated with treatment response, and that a high RDI contributed to longer survival [31]. This suggested that quantity of lenvatinib administration is strongly correlated with OS. Therefore, it is important to select patients who could continue lenvatinib.

Our study showed that CRP level could identify patients who were good candidates for lenvatinib treatment. In addition, this simple and readily available marker could predict TTF of lenvatinib. Our study showed that the RDI of a low CRP group was 0.93 and that of a high CRP group was 0.65 at 8 weeks. Sasaki et al. reported that RDI at 8 weeks was the most important predictive factor for OS [31]. They reported that the cut off value was 0.67. This is consistent with our findings. Low CRP group had better RDI at 8 weeks and had better OS. RDI at 8 weeks was a post-treatment predictor. However, the pre-treatment baseline CRP is very important when identifying suitable patients for lenvatinib treatment.

High CRP levels have been known to be associated with poor prognosis in patients with various malignancies, such as esophageal cancer, colorectal cancer, pancreatic cancer and renal cell carcinoma [12–15]. In regard to HCC, CRP level has been reported to be a useful prognostic factor in patients who underwent liver resection, transplantation, TACE and sorafenib treatment [17–22,27]. HCC patients with high CRP level had a poor prognosis and the optimal cutoff value of CRP was 1.0 mg/dL [18]. CRP is an acute phase reactant synthesized by hepatocytes and regulated by proinflammatory cytokines, especially IL-6. Sieghart et al. reported that CRP level was correlated with HCC staging [18]. In addition, elevated CRP level is associated with prognostic factors such as tumor size, vascular invasion, lymph node metastasis and distant metastasis [32]. This may suggest that CRP reflects tumor aggressiveness and systemic dissemination of cancer cells. Several mechanisms are proposed for CRP elevation in patients with malignancies. First, tumor growth induces tissue inflammation and increases CRP level [33]. Second, CRP is an indicator of an immune response to tumor antigens [34]. Third, tumor cells themselves increase the production of IL-6 [35]. These processes reflect CRP production. Furthermore, it is demonstrated that elevated CRP influences tumor progression [23]. Elevated IL-6 activates proliferation of cancer cells directly, accelerates angiogenesis and promotes binding to other organs [25]. Thus, IL-6 has a negative impact on the activity of cancer cells. HCC cells express IL-6 receptor [36]. And, the binding of IL-6 to its receptor induces the phosphorylation of Janus kinase 1 (JAK1), which is an upstream activator of STAT3. Activated STAT3 mediates the expression of genes involved with cell proliferation and promotes the cell cycle via expression of cyclins D1, D2 and D3 and c-Myc [37,38]. And, IL-6 increases angiogenesis by transcriptional upregulation of VEGF in JAK/STAT3 and hypoxia inducible factor 1α (HIF1α) dependent manner in cancer cells [39,40]. In addition, activated STAT3 plays an important role in metastasis to other organs. STAT3 activation induces overexpression of FGF, matrix metalloproteinase (MMP) and VEGF, which contribute to invasion and angiogenesis [41].

We think that CRP value suggests HCC status, independent of existing tumor stage and tumor marker. Furthermore, when HCC condition exceeded a certain point, general conditions getting worse. Therefore, HCC patients with high CRP value could take lenvatinib for smaller amount and shorter time. These conditions could lead to tumor progression and poor overall survival. The cut off value of CRP level is 1.0 mg/dL.

The present study has some limitations. First, this is a retrospective observational study. The number of this study subjects is small and the observational period is short. Therefore, a study with a larger sample size and longer observational period should be performed. Second, we could not clarify the relationship between CRP and IL-6. Because of retrospective study, storage tissue and sample are not exist. Third, not all patients could receive radiological evaluations. This may lead that CRP was an insignificant factor in multivariate analysis for TTP. Finally, this is a single arm study. Therefore, our results could only support that high CRP group was associated with poorer survival. However, we could not evaluate whether lenvatinib had no survival in high CRP group.

In conclusion, baseline CRP level was associated with OS, RDI, TTF and response to lenvatinib. High CRP level was a significant negative predictor of OS, independent of Child-Pugh score and existing tumor factors. Low serum CRP level, less than 1.0 mg/dL, is the best indicator of lenvatinib in patients with unresectable HCC.
